# Upregulation of cannabinoid receptor type 2, but not TSPO, in senescence-accelerated neuroinflammation in mice: a positron emission tomography study

**DOI:** 10.1186/s12974-019-1604-3

**Published:** 2019-11-10

**Authors:** Satoru Yamagishi, Yurika Iga, Masato Nakamura, Chika Takizawa, Dai Fukumoto, Takeharu Kakiuchi, Shingo Nishiyama, Hiroyuki Ohba, Hideo Tsukada, Kohji Sato, Yasuomi Ouchi

**Affiliations:** 1 Department of Organ and Tissue Anatomy, Hamamatsu University School of Medicine, 1-20-1 Handayama, Higashi-ku, Hamamatsu, 431-3192 Japan; 2 Central Research Laboratory, Hamamatsu Photonics KK, Hamamatsu, Japan; 3 Department of Biofunctional Imaging, Preeminent Medical Photonics Education & Research Center, Hamamatsu University School of Medicine, 1-20-1 Handayama, Higashi-ku, Hamamatsu, 431-3192 Japan

**Keywords:** Microglial activation, Senescence-accelerated prone mouse, Cannabinoid receptor type 2, Translocator protein, Positron emission tomography, Immunostaining

## Abstract

**Background:**

Microglial cells are activated in response to changes in brain homeostasis during aging, dementia, and stroke. Type 2 endocannabinoid receptors (CB2) and translocator protein 18 kD (TSPO) are considered to reflect distinct aspects of microglia-related neuroinflammatory responses in the brain. CB2 activation is considered to relate to the neuroprotective responses that may occur predominantly in the early stage of brain disorders such as Alzheimer’s disease, while an increase in TSPO expression tends to occur later during neuroinflammation, in a proinflammatory fashion. However, this information was deduced from studies with different animal samples under different experimental settings. In this study, we aimed to examine the early microglial status in the inflammation occurring in the brains of senescence-accelerated mouse prone 10 (SAMP10) mice, using positron emission tomography (PET) with CB2 and TSPO tracers, together with immunohistochemistry.

**Methods:**

Five- and 15-week-old SAMP10 mice that undergo neurodegeneration after 7 months of age were used. The binding levels of the TSPO tracer (*R*)-[^11^C]PK11195 and CB2 tracer [^11^C]NE40 were measured using PET in combination with immunohistochemistry for CB2 and TSPO. To our knowledge, this is the first study to report PET data for CB2 and TSPO at the early stage of cognitive impairment in an animal model.

**Results:**

The standard uptake value ratios (SUVRs) of [^11^C]NE40 binding were significantly higher than those of (*R*)-[^11^C]PK11195 binding in the cerebral cortical region at 15 weeks of age. At 5 weeks of age, the [^11^C]NE40 SUVR tended to be higher than the (*R*)-[^11^C]PK11195 SUVR. The (R)-[11C]PK11195 SUVR did not significantly differ between 5- and 15-week-old mice. Consistently, immunostaining analysis confirmed the upregulation of CB2, but not TSPO.

**Conclusions:**

The use of the CB2 tracer [^11^C]NE40 and/or an immunohistochemical approach allows evaluation of the role of microglia in acute neuroinflammatory processes in the early stage of neurodegeneration. The present results provide in vivo evidence of different responses of two types of microglia to senescence-accelerated neuroinflammation, implying the perturbation of microglial balance by aging. Specific treatment for CB2-positive microglia might help ameliorate senescence-related neuroinflammation and the following neurodegeneration.

## Background

Microglia change both morphologically and functionally from their resting state to their activated state in several neuroinflammatory and neurodegenerative diseases. Thus, microglial activation accompanies damage to the brain environment and is a reason why activated microglia represent an important marker of neuroinflammation [1]. Specifically, non-invasive imaging of activated microglia is a useful tool for detecting in vivo neuroinflammatory disease. To date, the first-generation translocator protein 18 kDa (TSPO) marker (*R*)-[^11^C]PK11195 has been widely used as a PET radioligand for this purpose. PET studies using (*R*)-[^11^C]PK11195 have been performed on patients with several neuroinflammatory diseases, including Alzheimer’s disease (AD) [2], Parkinson’s disease (PD) [3], and Huntington’s disease [4]. In addition, ischemic stroke causes direct insults to the brain tissue and the immediate activation of microglia, which can also be visualized by PET with (*R*)-[^11^C]PK11195 [5]. PET imaging provides an experimental advantage in that it allows studies on time-course changes in microglial activation. Specifically, as neuronal loss is a common feature of neuronal degeneration, acute neuronal damage, and aging, an early depiction of the extent to which the brain is compromised is important for treatment to delay the progression of such disease processes. In this context, techniques to image neuroinflammation in vivo, such as TSPO-PET, are helpful because no other imaging method exists to evaluate neuroinflammation in the living brain. It is currently well reported that the accumulation of (*R*)-[^11^C]PK11195 or the second-generation tracer [^18^F]DPA714 occurs relatively late after brain injury resulting from toxin injection [6] or traumatic insult [7, 8].

Endocannabinoid receptor type 2 (CB2) has recently been considered to be an alternative method for targeting activated microglia using PET imaging. The endocannabinoid system in the central nervous system has been shown to provide neuroprotective effects following brain injury [9, 10]. In particular, CB2 is upregulated in microglia during neurodegenerative and neuroinflammatory diseases such as AD [11–13], multiple sclerosis [14], PD [15], and ischemia [16–19], and is also known to be associated with microglial activity [20]. Moreover, the administration of a selective CB2 agonist reduced neuronal degeneration and improved cognitive function scores [11, 21]. Recently, a PET tracer that binds specifically to CB2 was developed to illustrate CB2 availability in vivo [22]. Considering that the neuroprotective role of microglia develops early in the course of brain illness, the failure to detect elevated [^11^C]NE40 uptake in AD patients [23] is understandable, possibly because the microglia activated during the chronic state of neurodegeneration are considered to be acting as proinflammatory cells [24] or because the demise of neurons that also express CB2 reduces detectable radioactivity. Recently, we reported that [^11^C]NE40 binding is increased in the peri-infarct area of the ipsilateral cortex at 24 h after induction of stroke, but not in the contralateral cortex, using a photothrombotic stroke model [25]. Following CB2 activation, uptake of a TSPO tracer is observed at the chronic stage, indicating that different inflammatory responses of microglia occur in the acute and chronic states after ischemia [26]. This early activation of CB2 might also be observed in a variety of animal models of neurological disorders such as AD, multiple sclerosis, and amyotrophic lateral sclerosis [27]. However, it is unclear whether CB2 is upregulated in animal models of senescence or early-stage mild cognitive impairment.

SAMPs (senescence-accelerated mouse prones) are inbred mouse lines showing accelerated aging. Of these, the SAMP10 line shows neuronal loss due to cortical degeneration in later life. It has been reported that SAMP10 mice show impaired conditioning memory with elevation of serum corticosterone at 12 weeks of age when they are chronically exposed to social isolation stress from 5 weeks of age [28]. In addition, they are likely to show significant reductions in cortical size from 7 months old because of neuronal loss [29]. In later stages at 8–16 months of age, not only the number of neurons, but also the length of dendrites and the spine density of cortical pyramidal neurons, are much reduced [30]. Behavioral changes related to neuronal dysfunction, such as decline of performance in passive avoidance and conditional avoidance tasks, also occurred in mice aged 10 to 12 months [29]. Interestingly, morphological impairments in microglia occurred at an early stage (~ 3 months old), preceding age-related neuronal degeneration [31], with the number of segments and tips and the combined lengths of microglial processes being much reduced. Hence, we aimed to investigate the status of microglia by comparing 5-week-old SAMP10 mice with 15-week-old animals, to detect early responses of microglia to brain aging.

In this study, we used PET to compare the levels of [^11^C]NE40 and (*R*)-[^11^C]PK11195 binding in the same living SAMP10 mice at different ages, because SAMP10 mice are suitable for evaluating the early response of microglia in terms of polarization around the timing of senescence-related microglial activation. The in vivo PET data were then corroborated by performing immunohistochemical analysis for CB2 and TSPO.

## Methods

### Animals

Eight 5-week-old and eight 15-week-old male senescence-accelerated mouse prone 10 (SAMP10) mice purchased from the SLC Company (Hamamatsu, Japan) were used in this study. The mice were housed with their littermates maximum of five animals in each cage with food and water available ad libitum. All animals were grouped in each cage. The fact that no individual animal was isolated did not allow evaluation of neuroinflammatory responses under social isolation in this study.

All animal protocols and the following experiments were approved by the ethics committees of the Central Research Laboratory at Hamamatsu Photonics and Hamamatsu University School of Medicine. In addition, all applicable institutional and/or national guidelines for the care and use of animals were followed.

### Tracer production

The synthesis of (*R*)-[^11^C]PK11195 was based on the literature [32, 33]. Briefly, [^11^C]CH_3_I was produced from [^11^C]CO_2_ or [^11^C]CH_4_ generated by irradiating N_2_ with protons in the presence of 0.2% O_2_ or 5% H_2_. Then, [^11^C]CH_3_I was reacted with desmethyl (*R*) PK11195 to form (*R*)-[^11^C]PK11195. The synthesis of [^11^C]NE40 was based on the literature [34]. Briefly, a helium stream containing [^11^C]CH_3_I was bubbled through 200 μl dimethylformamide containing 200 μg 2-oxo-7-hydroxy-8-butyloxy-1,2-dihydroquinoline-3-carboxylic acid cyclohexylamide and 2–4 mg Cs_2_CO_3_ [32–34].

### PET measurements

PET measurements were acquired on a high-resolution animal PET scanner (SHR-38000, Hamamatsu Photonics, Hamamatsu, Japan) with an axial field of view (FOV) of 330 mm, a transaxial FOV of 108 mm, and a transaxial spatial resolution of 2.3 mm in the center. Eight animals were scanned twice a day with (*R*)-[^11^C]PK11195 and [^11^C]NE40. A 2-h interval was left between the two scans, and the order of the scans was counterbalanced. The mice were anesthetized using 1.5–2.0% isoflurane in O_2_ for the duration of the entire imaging experiment. The mice were placed in the prone position on a fixation plate and then set within the gantry hole of the PET scanner. After a 15-min transmission scan utilizing an external ^68^Ge/^68^Ga rod source (67 MBq) for attenuation correction, a serial emission scan lasting for 60 min was performed immediately following each injection of (*R*)-[^11^C]PK11195 or [^11^C]NE40 tracer at a dose of 48 MBq/kg; the tracers were injected intravenously through a cannula inserted into the tail vein. The molar activity of each tracer was above 50 GBq/μmol. No arterial sampling was conducted. The PET data were reconstructed using 3D DRAMA (iteration 2, gamma 0.1) with a Gaussian filter of 1.0 mm full width at half maximum (FWHM), yielding a voxel size of 0.65 × 0.65 × 1.0167 mm for the reconstructed images. To obtain anatomical information, X-ray CT scans were performed immediately following the PET measurement, using a ClairvivoCT (Shimadzu Corporation, Kyoto, Japan).

### Data analysis and statistics

Using PMOD image analysis software (version 3.1; PMOD Technologies Ltd., Zurich, Switzerland), the standard uptake value ratios (SUVRs) for (*R*)-[^11^C]PK11195 and [^11^C]NE40 tracer binding were estimated by dividing the target SUV by the cerebellar SUV as the background level [35, 36]. The SUV was calculated as the measured radioactivity divided by the ratio of the total injected dose to the mouse body weight.

As described elsewhere [37, 38], elliptical regions of interest (ROIs) ranging from 12 to 24 mm^2^ in area were placed over the frontal cortex and hippocampus regions by referring to the X-ray CT images [39] (see Additional file 1: Figure S1).

One-way analysis of variance (ANOVA) was performed to compare the conditions (brain region, tracer uptake, and mouse age), with the significance level set at *p* < 0.05 with a correction for multiple comparisons. Within each age group, correlation analysis was conducted between the two tracer SUVRs ([^11^C]NE40 SUVR vs. (*R*)-[^11^C]PK11195 SUVR at either 5 or 15 weeks of age) with the Bonferroni correction for multiple correlations (*p* < 0.00625, 0.05/8), to examine deviations in the expression of these markers in the living brains of SAMP10 mice in relation to the progression of senescence.

### Immunohistochemistry

Immunostaining was performed as previously reported [40]. Briefly, mice were anesthetized with chloral hydrate (400 mg/kg) and then transcardially perfused with saline followed by 4% paraformaldehyde (PFA; pH 7.4). Their brains were removed, post-fixed in 4% PFA, and immersed in cryoprotectant solution (30% sucrose in phosphate-buffered saline [PBS]) until the tissue sank. Tissues were frozen in dry ice and stored at − 80 °C until use. Frozen coronal sections (20 μm thick) were cut using a cryostat. The slides were blocked with 10% donkey serum in PBS containing 0.1% Triton X-100 for 1 h at room temperature (RT) and then incubated overnight at 4 °C with primary antibodies. After washing, the slides were incubated for 1 h at RT with secondary antibodies. Then, they were washed three times with PBS and stained with DAPI to visualize the nuclei. Fluorescent images of a single focal plane were obtained by confocal microscopy (SP8, Leica, Wetzlar, Germany). The following primary antibodies were used in this study: goat anti-Iba1 (1:500, Abcam, Cambridge, UK), mouse anti-ATPB (1:200, Abcam), rabbit and mouse anti-cannabinoid receptor 2 (1:200, Cayman Chem, Ann Arbor, USA, and 1:200, Santa Cruz, Dallas, USA, respectively), rabbit anti-CaMKII (1:500, Abcam), mouse anti-GFAP (1:500, Merck Millipore, Burlington, USA), and rabbit anti-TSPO (1:200, Abcam). The secondary antibodies were as follows: Alexa Fluor 488 anti-rabbit IgG and anti-mouse IgG, Alexa Fluor 568 anti-rabbit IgG, Alexa Fluor 594 anti-mouse IgG, and Alexa Fluor 647 anti-goat IgG (1:500, Thermo Fisher Scientific, Waltham, USA).

## Results

### PET findings

**Fig. 1:**
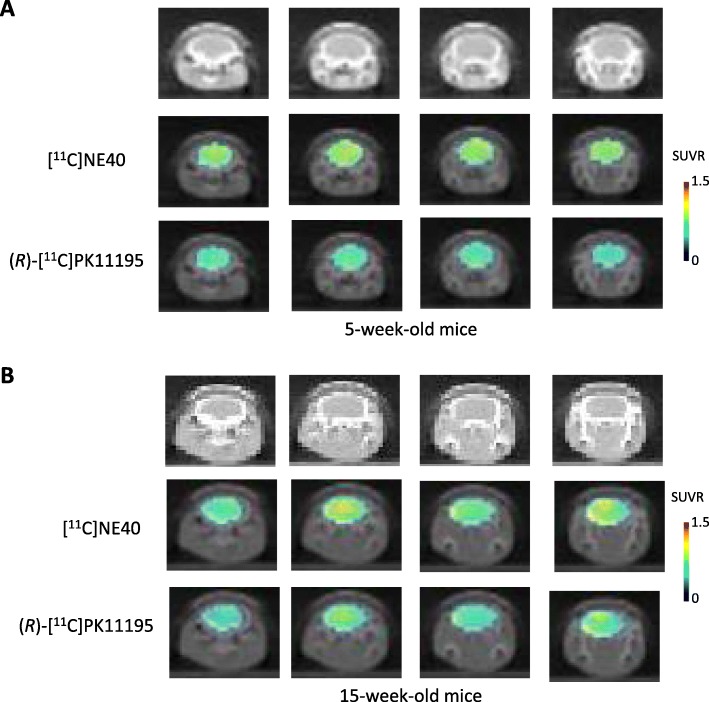
Coronal parametric PET images of [^11^C]NE40 and (*R*)-[^11^C]PK11195 tracers in 5-week-old (**a**) and 15-week-old (**b**) SAMP10 mice. The PET data are superimposed on X-ray CT images, and the color bar denotes the SUVR

**Fig. 2:**
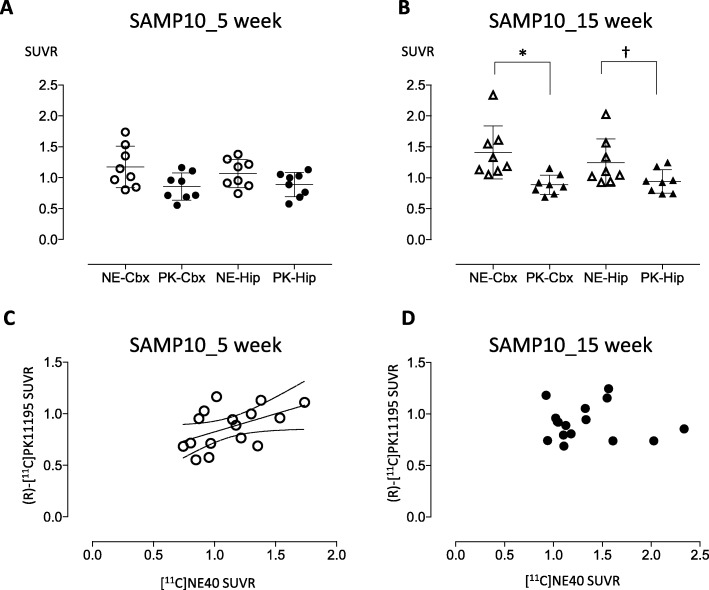
The SUVRs of [^11^C]NE40 and (*R*)-[^11^C]PK11195 SUVR (**a**, **b**) and their relationships (**c**, **d**). The [^11^C]NE40 SUVR was significantly higher than the (*R*)-[^11^C]PK11195 SUVR in the cerebral cortex in 15-week-old mice (**b**, *p < 0.05). The binding of the two tracers showed a positive correlation at 5 weeks of age (**c**). The dotted lines in **c** represent the 95% confidence intervals for the correlation. Dagger indicates a tendency for significant difference (*p* < 0.07)

Figure 1 shows the parametric PET images of [^11^C]NE40 and (*R*)-[^11^C]PK11195 uptake superimposed on CT images for 5-week-old (a) and 15-week-old (b) mice. The SUVRs of [^11^C]NE40 and (*R*)-[^11^C]PK11195 did not significantly differ in the cerebral cortex and hippocampus in 5-week-old SAMP10 mice (Figs. 1a and 2a, Table 1). By contrast, in 15-week-old SAMP10 mice, an ANOVA with multiple comparisons revealed a significantly higher SUVR for [^11^C]NE40 than for (*R*)-[^11^C]PK11195 in the frontal cortex (*F* = 5.062, *p* = 0.0119; Fig. 2b) and a tendency toward a higher [^11^C]NE40 SUVR in the hippocampus (*p* < 0.07; Figs. 1b and 2b, Table 1). The [^11^C]NE40 SUVR was not significantly higher in 15-week-old mice than in 5-week-old mice (Table 1, Additional file 1: Figure S1C).

**Table 1:** Differences in the levels of SUVR of [^11^C]NE40 and [^11^C] (*R*)PK11195

Age	[^11^C]NE40		[^11^C] (*R*)PK11195	
	Cerebral cortex	Hippocampus	Cerebral cortex	Hippocampus
5 weeks	1.18 ± 0.37	1.07 ± 0.23	0.85 ± 0.21	0.86 ± 0.21
15 weeks	1.41 ± 0.43*^a^	1.24 ± 0.38^b^	0.89 ± 0.16	0.94 ± 0.19

*p < 0.05 vs cerebral cortex [^11^C](*R*)PK11195 value (Tukey’s multiple comparisons test)

Tendency for significant difference (^a^*p* = 0.08, ^b^*p* = 0.10) vs 5-week-old mice data in [^11^C]NE40 measurement

Significant correlations were found between [^11^C]NE40 SUVRs and (*R*)-[^11^C]PK11195 SUVRs measured in the brains of 5-week-old mice (*r* = 0.501, *p* = 0.006; Fig. 2c), whereas no significant correlations were found in either 15-week-old SAMP10 mice (Fig. 2d) or 15-week-old SAMPR1 mice (*r* = 0.409, *p* = 0.117; Additional file 2: Figure S2B). In Additional file 2: Figure S2, data from 15-week-old SAMR1 mice (senescence-accelerated mouse resistant mice, the normal control for SAMP10 mice) are shown to help understand the changes seen in SAMP10 mice.

### Immunohistochemical findings

**Fig. 3:**
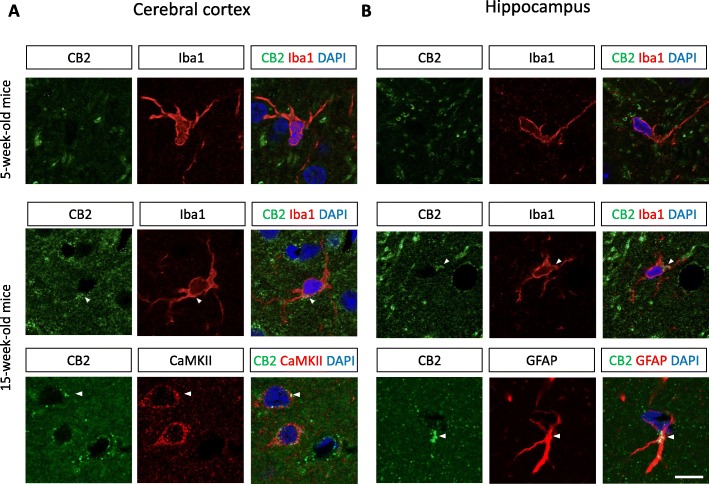
Double immunostaining for CB2 (green) and Iba1, CaMKII, or GFAP (red) in the cortex (**a**) and hippocampus (**b**) at 5 and 15 weeks of age. Note that the CB2 signal is greatly increased and localized to the microglia at 15 weeks of age. Arrowheads indicate CB2 signals in microglial cells. Scale bar 10 μm

Double immunostaining against CB2 and the microglial marker Iba1 at 5 weeks of age revealed CB2 immunopositive signals across the whole brain region, including the cerebral cortex and hippocampus (Fig. 3). The CB2 signals were not colocalized with Iba1 in the cortex and hippocampus, suggesting that CB2 is expressed on neurons and/or astrocytes. At 15 weeks, the intensity and number of CB2-positive signals were dramatically increased in the cortex and hippocampus of the SAMP10 mice (Fig. 3a and b, respectively). Interestingly, the CB2-positive signal was colocalized with Iba1+ microglia in both the cortex and hippocampus at 15 weeks, suggesting that the number of protective microglia increases in the early stage of senescence-related neurodegeneration. In addition, CB2-positive signals were observed in CAMKII+ neurons and GFAP+ astrocytes in the cortex and hippocampus, respectively. This result is consistent with the observations of the PET imaging using the [^11^C]NE40 tracer, as shown in Fig. 1b.

**Fig. 4:**
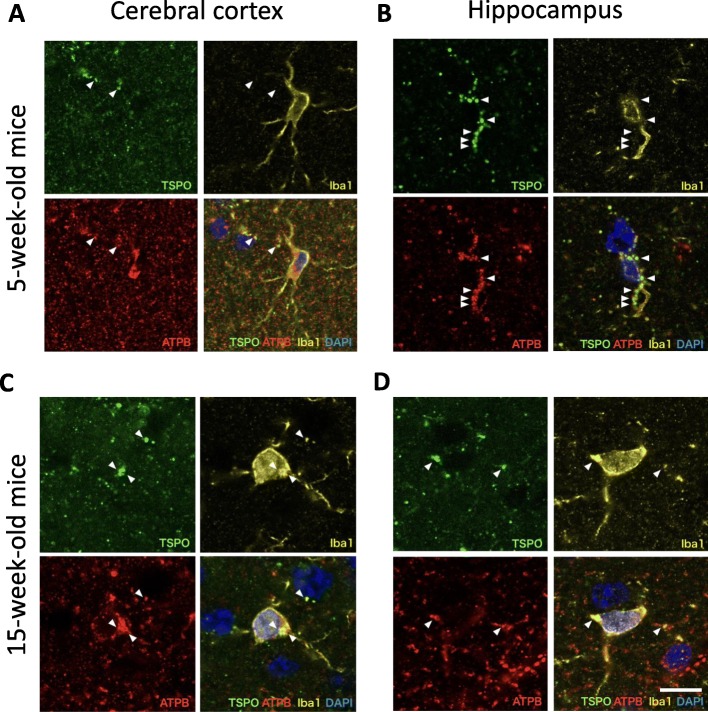
Triple immunostaining for TSPO (green), ATPB (red), and Iba1 (yellow) in the cortex (**a**, **c**) and hippocampus (**b**, **d**) areas at 5 (**a**, **b**) and 15 weeks of age (**c**, **d**). Arrowheads indicate TSPO signals in microglial cells. Scale bar 10 μm

In Fig. 4, triple immunostaining for TSPO (18 kDa), mitochondrial marker ATPB, and Iba1 shows detection of TSPO immunoreactive signal in the cerebral cortex and hippocampus at 5 weeks (Fig. 4a, b); hence, the TSPO signal is colocalized with ATPB and TSPO is located in the mitochondria at this age. The basal level of TSPO expression was mainly observed in the mitochondria of vascular endothelial cells, as previously reported (data not shown) [41]. Confocal imaging analysis revealed punctuate TSPO immunopositive staining along with Iba1+ cells in the hippocampus (Fig. 4b). However, this staining was not located inside or on the surface of the Iba1+ cells, but was localized to the contact sites of adjacent cells, indicating the accumulation of TSPO on the microglial facing side. In Fig. 4 c and d, TSPO signal can be detected inside the microglia in the cerebral cortex and hippocampus at 15 weeks. However, the overall intensity of the TSPO signal was not different to that in the 5-week-old mice and was consistent with the result of the PET imaging using the (*R*)-[^11^C]PK11195 tracer (Fig. 2b). This result suggests that the number of inflammatory microglia had not changed at this stage.

## Discussion

### Detection of microglial activation using the CB2 and TSPO tracers

In this study, we show that the SUVRs of [^11^C]NE40 were significantly higher than those of (*R*)-[^11^C]PK11195 in the cerebral cortical region in 15-week-old SAMP10 mice (Figs. 1 and 2b; Additional file 1: Figure S1C, D). Although there was no significant difference, the SUVRs of [^11^C]NE40 of 15-week-old mice tended to be higher than those of 5-week-old mice. Along similar lines, CB2 expression in microglia was increased in the cerebral cortex and hippocampus in the 15-week-old mice in comparison with the 5-week-old mice (Fig. 3a, b). However, (*R*)-[^11^C]PK11195 did not reveal significantly higher TSP binding in 15-week-old SAMP10 mice (Fig. 2b). To our knowledge, this is the first time that microglial activation has been shown in vivo using PET imaging of SAMP10 mice. SAMP10 mice show neuronal loss after 7 months and cognitive impairment from 12 months of age [29]. Prior to neurodegeneration, a morphological change to the microglia occurs in SAMP10 mice at 3 months of age [31]. Our observation of an increase in the protective microglial marker CB2 at this stage, but not in the inflammatory microglial marker TSPO, is indicative of the neuroinflammatory phenomena characteristic of the early stage of neuronal degeneration.

### Activation of protective microglia

The relatively rapid response of protective microglia extrapolated from the [^11^C]NE40 binding elevation suggests that the microglia try to maintain and/or recover the homeostasis of the brain environment; they may do this by engulfing degenerating neuronal debris or by releasing protective cytokines and/or neurotrophic factors such as interleukin (IL)-4, IL-10, BDNF, and IGF-1 [42]. However, these processes are not successful in the SAMP10 mice, and eventually, neuronal degeneration occurs, causing behavioral impairment and cognitive deficits. From the therapeutic point of view, the targeting of CB2 activation or other pharmacological treatments that can revitalize protective microglia might present new opportunities for therapies to prevent neuronal loss and cognitive impairment. As CB2 expression is limited to specific types of cells like microglia, and in this way differs from the broad expression of CB1 in the central nervous system, any psychoactive effect of cannabinoids should be minimal. In line with our hypothesis, administration of the selective agonist JWH-015 to CB2 significantly enhanced phagocytosis of Aβ_1-42_, which was inhibited by CD40 [43]. Additionally, the CD40 expression of microglial cells induced by IFN-γ was reduced by JWH-015 [43]. Furthermore, the CB1/CB2 mixed agonist WIN55,212-2 improved memory functions and decreased levels of inflammatory marker TNF-α and caspase-3 activation in an Aβ-induced neurodegeneration model in adult rats [44]. As SAMP10 mice exhibit senile amyloidosis at a late stage, these agonists might ameliorate their decline in cognitive function [45].

### TSPO and inflammatory microglia

TSPO is a highly inducible protein localized on the mitochondrial outer membrane upon microglial activation by inflammatory environment. In the healthy brain, endogenous TSPO expression is mainly observed in endothelial cells throughout the brain (data not shown) [41]. This endothelial expression was also observed by immunostaining in our SAMP10 mice at both 5 and 15 weeks of age. Therefore, the basal level of (*R*)-[^11^C]PK11195 binding should represent the endothelial expression of TSPO. We also observed TSPO signal that was non-overlapping with the mitochondrial outer-membrane marker ATPB, as previously reported, which suggests TSPO localization in subcellular organelles other than mitochondria [46]. It is also known that, upon inflammation, TSPO is expressed not only in microglia, but also in astrocytes [47, 48]. In the SAMP10 mice, as the TSPO level was not enhanced at 15 weeks, it is probable that the level of TSPO in astrocytes was also not enhanced. The inflammatory IL-6 level is reportedly increased at 14 months; therefore, TSPO would probably be upregulated at the later stage of brain degeneration [49]. This speculation is consistent with our previous finding that uptake of (*R*)-[^11^C]PK11195 is high at a later period after stroke in a rat model [35].

In the chronic stage of inflammation [6], neurotoxic M1-phenotype microglia are dominant, and the TSPO tracer (*R*)-[^11^C]PK11195 reflects inflammatory rather than anti-inflammatory microglial activity [24]. In support of this assertion, we showed an elevation in the second-generation TSPO tracer [^11^C]DPA713 for illustrating chronically activated microglia under inflammatory circumstances [50]. We recently used a photochemically induced thrombosis (PIT) model to show that the CB2 response occurs earlier than the TSPO response [25]. This suggests that the activation of protective microglia is the key event and that this occurs prior to inflammatory microglial activation in the case of neuronal damage.

### Limitations

This study is subject to a number of limitations. First, we measured changes in the uptake of both [^11^C]NE40 and (*R*)-[^11^C]PK11195 in 5- and 15-week-old SAMP10 mice. These data do not allow us to elucidate the timing of the state switch, dominancy, protective activity, or inflammatory processes of microglia. Later stage SAMP10 mice might generate different outcomes with regard to inflammatory substances and cells [49], and further study is needed to address changes in the time course of inflammatory events during senescence-related neuronal loss. Second, because the spatial resolution of the PET scanner used was only 2.3 mm, the results may be subject to partial volume effects. To reduce such effects, we tried to set the measurement ROIs to twice the size of the FWHM of the scanner. An alternative would be to use autoradiography instead of PET, although measures of parameter changed in the same animal cannot be obtained using autoradiography. In this study, we therefore used the cerebellum as a non-specific region for CB2/TSPO expression to estimate the specific binding of the tracers, which is a widely accepted method.

## Conclusion

The detected increase in [^11^C]NE40 binding but not (*R*)-[^11^C]PK11195 binding in 15-week-old SAMP10 mice, which was concomitant with a higher CB2 immunochemical expression within the microglia, indicates that acutely activated microglia may be involved in a neuroprotective process of neuroinflammation in the early stage of senescence-related neuronal loss. While the new CB2 tracer [^11^C]A836339 has been reported to exceed the sensitivity of [^11^C]NE40 for in vivo binding to CB2 under a chronic state of neurodegeneration [13], the present results suggest that [^11^C]NE40 might be adequate for depicting activated microglia in the very early stage of brain disorders.

Our results provide in vivo evidence of different responses of two types of microglia to senescence-accelerated neuroinflammation, implying the perturbation of microglial balance by aging. Specific treatment that stimulates CB2-positive microglia or expression of CB2 in microglia to promote neuroprotection might help ameliorate senescence-related neuroinflammation.

## Supplementary information

**Additional file 1: Figure S1.** CT images and regions of interest in the cerebral cortex (A, yellow, approximately −1 mm from the bregma) and hippocampus (B, red, approximately −2 mm from the bregma). The PET data are rearranged according to each tracer to demonstrate the changes across all groups (C, D). Cbx: cerebral cortex, Hip: hippocampus; 5w: 5 weeks of age; 15w: 15 weeks of age. †a tendency (*p*=0.08) vs 5-week-old mice data in [^11^C]NE40 measurement.

**Additional file 2: Figure S2.** The SUVRs of the two tracers did not significantly differ in 15-week-old SAMR1 mice (A). The correlation between the SUVRs of the two tracers failed to reach statistical significance (*p* = 0.117, *r* = 0.409) (B). [^11^C]NE40 uptake was found significantly higher in 15-week old SAMP10 mice than in 15-week-old SAMR1 mice.

## Data Availability

The datasets supporting the conclusions of this article are available by request but will not be posted on a repository at this point due to intellectual property/confidentiality issues.

## Abbreviations

(*R*)-[^11^C]PK11195: (*R*)-N-(11) C-methyl-N-(1-methylpropyl)-1(2-chlorophenyl) isoquinoline-3-carboxamide
^11^C-DPA713: N,N-diethyl-2-[2-(4-methoxyphenyl)-5,7-dimethylpyrazolo [1,5-a]pyrimidin-3-yl] acetamide
^11^C-NE40: 2-oxo-7-[11C]methoxy-8butyloxy-1,2-dihydroquinoline-3-carboxylic acid cyclohexylamide
AD: Alzheimer’s disease
CB2: Type 2 endocannabinoid receptor
FWHM: Full width at half maximum
PET: Positron emission tomography
PFA: Paraformaldehyde
R1: Tracer influx index
RT: Room temperature
SAMP: Senescence-accelerated mouse prone
SUVR: Standard uptake value ratio
TSPO: Translocator protein

## References

[1] Venneti S, Wiley CA, Kofler J (2009). Imaging microglial activation during neuroinflammation and Alzheimer’s disease. J NeuroImmune Pharmacol.

[2] Yokokura M, Mori N, Yagi S, Yoshikawa E, Kikuchi M, Yoshihara Y, Wakuda T, Sugihara G, Takebayashi K, Suda S (2011). In vivo changes in microglial activation and amyloid deposits in brain regions with hypometabolism in Alzheimer’s disease. Eur J Nucl Med Mol Imaging.

[3] Ouchi Y, Yoshikawa E, Sekine Y, Futatsubashi M, Kanno T, Ogusu T, Torizuka T (2005). Microglial activation and dopamine terminal loss in early Parkinson’s disease. Ann Neurol.

[4] Venneti S, Lopresti BJ, Wiley CA (2006). The peripheral benzodiazepine receptor (translocator protein 18kDa) in microglia: from pathology to imaging. Prog Neurobiol.

[5] Pappata S, Levasseur M, Gunn RN, Myers R, Crouzel C, Syrota A, Jones T, Kreutzberg GW, Banati RB (2000). Thalamic microglial activation in ischemic stroke detected in vivo by PET and [11C]PK11195. Neurology.

[6] Shukuri M, Takashima-Hirano M, Tokuda K, Takashima T, Matsumura K, Inoue O, Doi H, Suzuki M, Watanabe Y, Onoe H (2011). In vivo expression of cyclooxygenase-1 in activated microglia and macrophages during neuroinflammation visualized by PET with 11C-ketoprofen methyl ester. J Nucl Med.

[7] Wang Y, Yue X, Kiesewetter DO, Niu G, Teng G, Chen X (2014). PET imaging of neuroinflammation in a rat traumatic brain injury model with radiolabeled TSPO ligand DPA-714. Eur J Nucl Med Mol Imaging.

[8] Israel I, Ohsiek A, Al-Momani E, Albert-Weissenberger C, Stetter C, Mencl S, Buck AK, Kleinschnitz C, Samnick S, Siren AL (2016). Combined [(18) F]DPA-714 micro-positron emission tomography and autoradiography imaging of microglia activation after closed head injury in mice. J Neuroinflammation.

[9] Braun M, Khan ZT, Khan MB, Kumar M, Ward A, Achyut BR, Arbab AS, Hess DC, Hoda MN, Baban B (2018). Selective activation of cannabinoid receptor-2 reduces neuroinflammation after traumatic brain injury via alternative macrophage polarization. Brain Behav Immun.

[10] Magid L, Heymann S, Elgali M, Avram L, Cohen Y, Liraz-Zaltsman S, Mechoulam R, Shohami E (2019). Role of CB2 receptor in the recovery of mice after traumatic brain injury. J Neurotrauma.

[11] Martin-Moreno AM, Reigada D, Ramirez BG, Mechoulam R, Innamorato N, Cuadrado A, de Ceballos ML (2011). Cannabidiol and other cannabinoids reduce microglial activation in vitro and in vivo: relevance to Alzheimer’s disease. Mol Pharmacol.

[12] Savonenko AV, Melnikova T, Wang Y, Ravert H, Gao Y, Koppel J, Lee D, Pletnikova O, Cho E, Sayyida N (2015). Cannabinoid CB2 receptors in a mouse model of Abeta amyloidosis: immunohistochemical analysis and suitability as a PET biomarker of neuroinflammation. PLoS One.

[13] Wu J, Bie B, Yang H, Xu JJ, Brown DL, Naguib M (2013). Activation of the CB2 receptor system reverses amyloid-induced memory deficiency. Neurobiol Aging.

[14] Palazuelos J, Davoust N, Julien B, Hatterer E, Aguado T, Mechoulam R, Benito C, Romero J, Silva A, Guzman M (2008). The CB (2) cannabinoid receptor controls myeloid progenitor trafficking: involvement in the pathogenesis of an animal model of multiple sclerosis. J Biol Chem.

[15] Concannon RM, Okine BN, Finn DP, Dowd E (2015). Differential upregulation of the cannabinoid CB2 receptor in neurotoxic and inflammation-driven rat models of Parkinson’s disease. Exp Neurol.

[16] Ashton JC, Rahman RM, Nair SM, Sutherland BA, Glass M, Appleton I (2007). Cerebral hypoxia-ischemia and middle cerebral artery occlusion induce expression of the cannabinoid CB2 receptor in the brain. Neurosci Lett.

[17] Fernandez-Lopez D, Faustino J, Derugin N, Wendland M, Lizasoain I, Moro MA, Vexler ZS (2012). Reduced infarct size and accumulation of microglia in rats treated with WIN 55,212-2 after neonatal stroke. Neuroscience.

[18] Zarruk JG, Fernandez-Lopez D, Garcia-Yebenes I, Garcia-Gutierrez MS, Vivancos J, Nombela F, Torres M, Burguete MC, Manzanares J, Lizasoain I, Moro MA (2012). Cannabinoid type 2 receptor activation downregulates stroke-induced classic and alternative brain macrophage/microglial activation concomitant to neuroprotection. Stroke.

[19] Zhang M, Martin BR, Adler MW, Razdan RK, Jallo JI, Tuma RF (2007). Cannabinoid CB2 receptor activation decreases cerebral infarction in a mouse focal ischemia/reperfusion model. J Cereb Blood Flow Metab.

[20] Benito C, Tolon RM, Pazos MR, Nunez E, Castillo AI, Romero J (2008). Cannabinoid CB2 receptors in human brain inflammation. Br J Pharmacol.

[21] Ramirez BG, Blazquez C, Gomez del Pulgar T, Guzman M, de Ceballos ML (2005). Prevention of Alzheimer’s disease pathology by cannabinoids: neuroprotection mediated by blockade of microglial activation. J Neurosci.

[22] Evens N, Vandeputte C, Coolen C, Janssen P, Sciot R, Baekelandt V, Verbruggen AM, Debyser Z, Van Laere K, Bormans GM (2012). Preclinical evaluation of [11C]NE40, a type 2 cannabinoid receptor PET tracer. Nucl Med Biol.

[23] Postnov A, Ahmad R, Evens N, Versijpt J, Vandenbulcke M, Yaqub M, Verbruggen A, Bormans G, Vandenberghe W, Laere K (2013). Quantification of 11C-NE40, a novel PET radioligand for CB2 receptor imaging. J Nucl Med.

[24] Banati RB, Newcombe J, Gunn RN, Cagnin A, Turkheimer F, Heppner F, Price G, Wegner F, Giovannoni G, Miller DH (2000). The peripheral benzodiazepine binding site in the brain in multiple sclerosis - quantitative in vivo imaging of microglia as a measure of disease activity. Brain.

[25] Hosoya T, Fukumoto D, Kakiuchi T, Nishiyama S, Yamamoto S, Ohba H, Tsukada H, Ueki T, Sato K, Ouchi Y (2017). In vivo TSPO and cannabinoid receptor type 2 availability early in post-stroke neuroinflammation in rats: a positron emission tomography study. J Neuroinflammation.

[26] Walter HL, Walberer M, Rueger MA, Backes H, Wiedermann D, Hoehn M, Neumaier B, Graf R, Fink GR, Schroeter M (2015). In vivo analysis of neuroinflammation in the late chronic phase after experimental stroke. Neuroscience.

[27] Ni R, Mu L, Ametamey S (2019). Positron emission tomography of type 2 cannabinoid receptors for detecting inflammation in the central nervous system. Acta Pharmacol Sin.

[28] Chida Y, Sudo N, Mori J, Kubo C (2006). Social isolation stress impairs passive avoidance learning in senescence-accelerated mouse (SAM). Brain Res.

[29] Shimada A (1999). Age-dependent cerebral atrophy and cognitive dysfunction in SAMP10 mice. Neurobiol Aging.

[30] Shimada A, Tsuzuki M, Keino H, Satoh M, Chiba Y, Saitoh Y, Hosokawa M (2006). Apical vulnerability to dendritic retraction in prefrontal neurones of ageing SAMP10 mouse: a model of cerebral degeneration. Neuropathol Appl Neurobiol.

[31] Hasegawa-Ishii S, Takei S, Chiba Y, Furukawa A, Umegaki H, Iguchi A, Kawamura N, Yoshikawa K, Hosokawa M, Shimada A (2011). Morphological impairments in microglia precede age-related neuronal degeneration in senescence-accelerated mice. Neuropathology.

[32] Hashimoto K, Inoue O, Suzuki K, Yamasaki T, Kojima M (1989). Synthesis and evaluation of 11C-PK 11195 for in vivo study of peripheral-type benzodiazepine receptors using positron emission tomography. Ann Nucl Med.

[33] Cremer JE, Hume SP, Cullen BM, Myers R, Manjil LG, Turton DR, Luthra SK, Bateman DM, Pike VW (1992). The distribution of radioactivity in brains of rats given [N-methyl-11C] PK 11195 in vivo after induction of a cortical ischaemic lesion. Int J Rad Appl Instrum B.

[34] Evens N, Muccioli GG, Houbrechts N, Lambert DM, Verbruggen AM, Van Laere K, Bormans GM (2009). Synthesis and biological evaluation of carbon-11- and fluorine-18-labeled 2-oxoquinoline derivatives for type 2 cannabinoid receptor positron emission tomography imaging. Nucl Med Biol.

[35] Fukumoto D, Hosoya T, Nishiyama S, Harada N, Iwata H, Yamamoto S, Tsukada H (2011). Multiparametric assessment of acute and subacute ischemic neuronal damage: a small animal positron emission tomography study with rat photochemically induced thrombosis model. Synapse.

[36] Martin A, Vazquez-Villoldo N, Gomez-Vallejo V, Padro D, Soria FN, Szczupak B, Plaza-Garcia S, Arrieta A, Reese T, Llop J (2016). In vivo imaging of system xc- as a novel approach to monitor multiple sclerosis. Eur J Nucl Med Mol Imaging.

[37] Ouchi Y, Tsukada H, Kakiuchi T, Nishiyama S, Futatsubashi M (1998). Changes in cerebral blood flow and postsynaptic muscarinic cholinergic activity in rats with bilateral carotid artery ligation. J Nucl Med.

[38] Shimizu Y, Yamamoto S, Fukumoto D, Ohba H, Kakiuchi T, Nishiyama S, Yoshikawa E, Tsukada H, Okada H, Ouchi Y. Loud noise exposure during activity and neurogenesis in the living rat brain: preliminary study. J Neurol Neurophysiol. 2014;5:100253.

[39] Levine JM (1994). Increased expression of the NG2 chondroitin-sulfate proteoglycan after brain injury. J Neurosci.

[40] Yamagishi S, Yamada K, Sawada M, Nakano S, Mori N, Sawamoto K, Sato K. Netrin-5 is highly expressed in neurogenic regions of the adult brain. Front Cell Neurosci. 2015;9:146. 10.3389/fncel.2015.00146 PMC4403520 25941474

[41] Betlazar Calina, Harrison-Brown Meredith, Middleton Ryan, Banati Richard, Liu Guo-Jun (2018). Cellular Sources and Regional Variations in the Expression of the Neuroinflammatory Marker Translocator Protein (TSPO) in the Normal Brain. International Journal of Molecular Sciences.

[42] Neumann H, Kotter MR, Franklin RJM (2008). Debris clearance by microglia: an essential link between degeneration and regeneration. Brain.

[43] Ehrhart J, Obregon D, Mori T, Hou H, Sun N, Bai Y, Klein T, Fernandez F, Tan J, Shytle RD (2005). Stimulation of cannabinoid receptor 2 (CB2) suppresses microglial activation. J Neuroinflammation.

[44] Fakhfouri G, Ahmadiani A, Rahimian R, Grolla AA, Moradi F, Haeri A (2012). WIN55212-2 attenuates amyloid-beta-induced neuroinflammation in rats through activation of cannabinoid receptors and PPAR-gamma pathway. Neuropharmacology.

[45] Higuchi K (1997). Genetic characterization of senescence-accelerated mouse (SAM). Exp Gerontol.

[46] Hardwick M, Fertikh D, Culty M, Li H, Vidic B, Papadopoulos V (1999). Peripheral-type benzodiazepine receptor (PBR) in human breast cancer: correlation of breast cancer cell aggressive phenotype with PBR expression, nuclear localization, and PBR-mediated cell proliferation and nuclear transport of cholesterol. Cancer Res.

[47] Notter T, Coughlin JM, Gschwind T, Weber-Stadlbauer U, Wang Y, Kassiou M, Vernon AC, Benke D, Pomper MG, Sawa A, Meyer U (2018). Translational evaluation of translocator protein as a marker of neuroinflammation in schizophrenia. Mol Psychiatry.

[48] Notter T, Coughlin JM, Sawa A, Meyer U (2018). Reconceptualization of translocator protein as a biomarker of neuroinflammation in psychiatry. Mol Psychiatry.

[49] Kumagai N, Chiba Y, Hosono M, Fujii M, Kawamura N, Keino H, Yoshikawa K, Ishii S, Saitoh Y, Satoh M (2007). Involvement of pro-inflammatory cytokines and microglia in an age-associated neurodegeneration model, the SAMP10 mouse. Brain Res.

[50] Yokokura M, Terada T, Bunai T, Nakaizumi K, Takebayashi K, Iwata Y, Yoshikawa E, Futatsubashi M, Suzuki K, Mori N, Ouchi Y (2017). Depiction of microglial activation in aging and dementia: positron emission tomography with [(11) C]DPA713 versus [(11) C]( R)PK11195. J Cereb Blood Flow Metab.

