# Supplementary figures and images for: Upregulation of cannabinoid receptor type 2, but not TSPO, in senescence-accelerated neuroinflammation in mice: a positron emission tomography study
Source: J Neuroinflammation. 2019 Nov 10;16:208. doi: 10.1186/s12974-019-1604-3 (PMC6842455; doi:10.1186/s12974-019-1604-3)

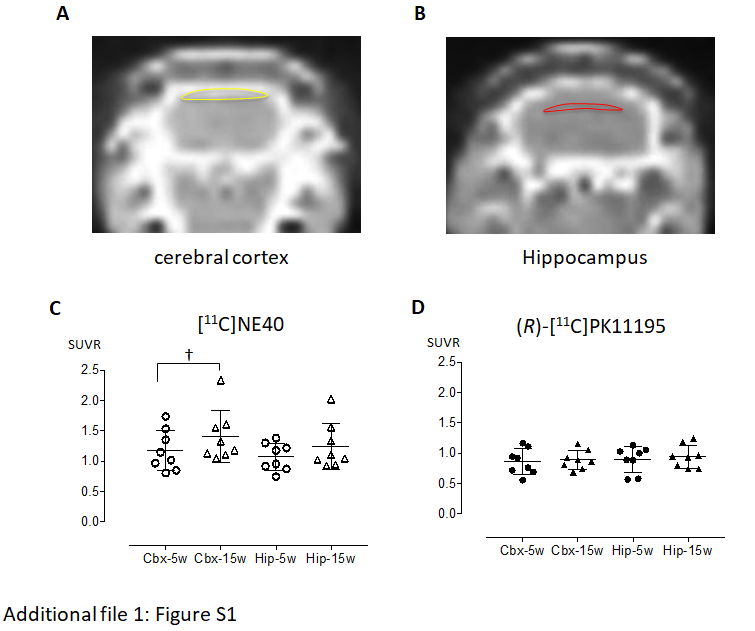

Supplement: Supplementary file 1 — Additional file 1: Figure S1. CT images and regions of interest in the cerebral cortex (A, yellow, approximately −1 mm from the bregma) and hippocampus (B, red, approximately −2 mm from the bregma). The PET data are rearranged according to each tracer to demonstrate the changes across all groups (C, D). Cbx: cerebral cortex, Hip: hippocampus; 5w: 5 weeks of age; 15w: 15 weeks of age. †a tendency (p=0.08) vs 5-week-old mice data in [11C]NE40 measurement. [file 12974_2019_1604_MOESM1_ESM.png]

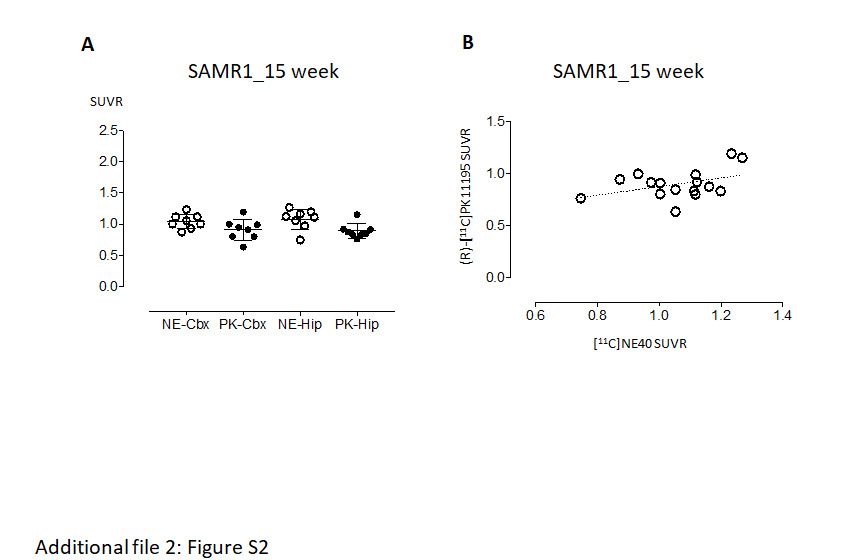

Supplement: Supplementary file 2 — Additional file 2: Figure S2. The SUVRs of the two tracers did not significantly differ in 15-week-old SAMR1 mice (A). The correlation between the SUVRs of the two tracers failed to reach statistical significance (p = 0.117, r = 0.409) (B). [11C]NE40 uptake was found significantly higher in 15-week old SAMP10 mice than in 15-week-old SAMR1 mice. [file 12974_2019_1604_MOESM2_ESM.png]
